# The microbiology of amniotic fluid sludge: a systematic review and meta-analysis

**DOI:** 10.1007/s00404-026-08492-2

**Published:** 2026-06-19

**Authors:** Elena Shipitsyna, Olga Pachulia, Vladislava Khalenko, Ekaterina Kopteeva, Anastasiya Sazonova, Natalya Zhestkova, Olesya Bespalova

**Affiliations:** 1 Department of Medical Microbiology, D.O. Ott Research Institute of Obstetrics, Gynecology and Reproductive Medicine, St. Petersburg, Russia; 2 Department of Obstetrics and Perinatology, D.O. Ott Research Institute of Obstetrics, Gynecology and Reproductive Medicine, St. Petersburg, Russia

**Keywords:** Amniotic fluid sludge, Intra-amniotic infection, Ureaplasma spp., Amniocentesis, Meta-analysis

## Abstract

**Purpose:**

Amniotic fluid sludge (AFS) is a sonographic finding associated with intra-amniotic infection and spontaneous preterm birth. The microbial composition of AFS remains poorly characterized. This systematic review and meta-analysis aimed to consolidate existing data on microbial prevalence in AFS and identify consistent microbial patterns.

**Methods:**

A systematic search of MEDLINE (PubMed), Dimensions, and OpenAlex was conducted from inception to February 11, 2026. Studies reporting the prevalence of microorganisms in AFS (minimum 10 cases) from pregnant women with AFS diagnosed via transvaginal ultrasound were included. Two reviewers independently performed screening, data extraction, and risk-of-bias assessment, with a third reviewer resolving discrepancies. Random-effects meta-analysis of proportions was used to pool overall and pathogen-specific prevalence.

**Results:**

Four studies comprising 85 women met inclusion criteria. The pooled prevalence of any microorganism was 35% (95%CI 22–48%), with moderate heterogeneity (I^2^ = 36.8%). *Ureaplasma* spp. were the most frequently detected pathogens (pooled prevalence 17%, 95%CI 8–27%). Sensitivity analysis revealed that sampling technique was a source of heterogeneity in this dataset; excluding the single study using transvaginal amniocentesis reduced the pooled prevalence to 28% (95%CI 17–41%) and eliminated statistical heterogeneity (I^2^ = 0.0%). Transvaginally collected samples exhibited significantly greater microbial diversity (median 2 vs. 1 microorganism/sample, *p* = 0.005) and contained vaginal commensals, suggesting possible contamination.

**Conclusion:**

In transabdominal cohorts, the pooled prevalence of microorganisms (any) in amniotic fluid sludge was 28% (95%CI 17–41%), with *Ureaplasma* spp. emerging as the predominant pathogen when an infectious agent was identified. Transvaginal amniocentesis resulted in greater microbial diversity and detection of vaginal commensals, consistent with contamination. Our findings suggest that transabdominal sampling should be the preferred methodological approach for future studies of AFS microbiology.

**Supplementary Information:**

The online version contains supplementary material available at https://doi.org/10.1007/s00404-026-08492-2.

## What does this study adds to the clinical work

**Table:** 

This meta-analysis demonstrates that detectable microorganisms are present in approximately 28% of amniotic fluid sludge (AFS) cases in transabdominal cohorts, with *Ureaplasma* spp. identified as the predominant pathogen. It demonstrates that transvaginal sampling is associated with greater microbial diversity and detection of vaginal commensals, suggesting possible contamination, and that transabdominal amniocentesis may be the preferred methodological approach for the evaluation of AFS microbiology. The findings highlight that over two-thirds of AFS cases remain "sterile," pointing to the importance of considering non-bacterial triggers and potential immunomodulatory management.	

## Introduction

Amniotic fluid sludge (AFS) is a recognized sonographic finding, appearing as particulate, echogenic material in the amniotic fluid, typically located in close proximity to the internal cervical os. It is widely acknowledged as a significant clinical marker associated with an increased risk of adverse pregnancy outcomes, most notably spontaneous preterm birth (PTB), preterm prelabor rupture of membranes (PPROM), chorioamnionitis, and early onset neonatal sepsis [1]. Its visual presence on transvaginal ultrasound is interpreted as a potential sign of intra-amniotic infection and/or inflammation, even in asymptomatic patients with a sonographically short cervix.

Importantly, the clinical significance of sonographically detected particulate matter depends critically on gestational age. Before 33 weeks, this material is predominantly pus-like and strongly associated with intra-amniotic infection, acute histologic chorioamnionitis, and funisitis. At term, particulate matter typically represents vernix or meconium, reflecting normal fetal maturation rather than pathology [2]. Thus, AFS transitions from a pathological sign of infection in preterm pregnancies to a physiological finding at term.

AFS is thought to result from ascending infection, which leads to microbial invasion of the amniotic cavity. This triggers a strong inflammatory response, recruiting inflammatory cells (primarily neutrophils) that aggregate with microorganisms to form the particulate matter characteristic of AFS [3]. Some evidence points to AFS as a microbial biofilm, a community of microorganisms embedded in a self-produced matrix of extracellular polymeric substances [4]. Understanding the precise microbial composition of AFS is crucial, as it may directly inform the pathogenesis of PTB, guide targeted antimicrobial therapy and refine risk stratification for clinical management.

Despite diverse methodological approaches—including culture and molecular techniques—to identify microorganisms in AFS cases, the literature remains fragmented and inconsistent. The microbial profiles in AFS are characterized by considerable heterogeneity, comprising a diverse array of microorganisms. Many of these are commensals of the lower genital tract. Most frequently reported species include *Ureaplasma* spp. (*U. parvum* and *U. urealyticum*), *Mycoplasma hominis*, *Candida albicans*, *Streptococcus* spp., and *Fusobacterium nucleatum* [2, 3]; however, their reported prevalence varies markedly across studies. This lack of accurate data hinders the development of effective clinical management strategies, including targeted antimicrobial treatment, risk stratification, and pregnancy surveillance protocols. To address this gap, we performed a systematic review and meta-analysis aiming to consolidate existing data, provide the first pooled estimates of microbial prevalence in AFS, and identify consistent microbial patterns associated with this sonographic finding.

## Methods

### Study design and search strategy

This systematic review and meta-analysis was designed to identify all relevant studies investigating the prevalence of microorganisms in amniotic fluid in women with AFS and to calculate pooled prevalence estimates for overall and pathogen-specific microbial detection. Given the anticipated limited number of studies and small sample sizes in the published literature on AFS microbiology, we first conducted an exploratory assessment to determine whether sufficient data existed to support a meaningful meta-analysis. After confirming that pooled estimates could be generated and would yield interpretable results, we proceeded with a full systematic review and meta-analysis in accordance with PRISMA 2020 guidelines [5]. The completed PRISMA 2020 checklist is provided in Table S1. A predefined internal protocol with strict inclusion criteria (e.g., minimum 10 AFS cases) guided the review. However, due to this preliminary feasibility phase, the review was not prospectively registered in PROSPERO.

A systematic literature search was performed across MEDLINE (via PubMed), Dimensions, and OpenAlex to identify relevant articles published from inception up to February 11, 2026. The search was restricted to studies published in English or those providing a detailed English abstract for screening. The search strategy employed the following keywords and Boolean operators: (1) MEDLINE: ("amniotic fluid sludge"[tiab] OR "intra-amniotic sludge"[tiab]) AND (infectio*[tiab] OR micro*[tiab] OR biofilm[tiab]); and (2) Dimensions/OpenAlex: ("amniotic fluid sludge" OR "intra-amniotic sludge") AND (infection OR microorganisms OR biofilm).

In addition to the primary database search, the artificial intelligence (AI)-enhanced academic search engine Google Scholar Labs was used to assist in identifying potentially relevant studies, prompted to "find papers on the microorganisms found in women with amniotic fluid sludge." All AI-generated results were critically reviewed and cross-verified against primary database searches by the authors; no studies were included based solely on AI-driven results. Finally, the search was supplemented by manual screening of reference lists from retrieved full-text articles and relevant reviews to identify any additional studies not captured by the electronic search.

### Eligibility criteria

Studies were selected based on the following inclusion criteria: (1) population: pregnant women with a diagnosis of AFS identified via transvaginal ultrasound; (2) exposure: assessment of the prevalence of microorganisms (any) or specific microorganisms in the AFS or amniotic fluid samples; (3) outcome: studies reporting sufficient data to calculate a prevalence (number of cases and total sample size); (4) sample size threshold: to ensure statistical reliability, studies were required to include a minimum of 10 AFS cases investigated for microorganisms; (5) study design: observational studies (prospective or retrospective cohort studies, cross-sectional studies, case series) that report prevalence data.

Exclusion criteria included: (1) reviews, editorials, case reports, dissertations, conference reports without full data; (2) studies not using standard microbiological methods (culture or molecular methods like PCR) for detection; (3) studies that did not report raw prevalence data for microorganisms in AFS.

### Study selection and data extraction

Two independent reviewers (ES, OP) screened titles and abstracts, followed by a full-text review of potentially eligible articles. Disagreements were resolved through discussion with a third reviewer (VK). A PRISMA flow diagram documented the study selection process. Data were extracted using a standardized, pre-piloted form. Extracted data included: (1) study characteristics (author, year, country, study design, sample size, population characteristics, gestational age at admission or sampling); (2) microbiological methods used (culture, PCR, microorganisms identified); (3) number of participants with AFS, number of cases with identified microorganisms, and the proportion of positive samples. To facilitate data synthesis and transparency, an individual participant-level data matrix was constructed detailing the presence of specific microorganisms, the type of amniocentesis performed, microbiological detection methods used for each study sample, and the gestational age at admission or sampling.

### Risk-of-bias assessment

The methodological quality and risk of bias of the included studies were assessed independently by two reviewers (ES, OP) using the Joanna Briggs Institute (JBI) critical appraisal checklist for prevalence studies [6]. The checklist comprises nine items designed to evaluate: (1) appropriateness of the sampling frame; (2) appropriateness of the sampling technique; (3) adequacy of the sample size; (4) description of study subjects and setting; (5) sufficiency of data analysis coverage; (6) validity of methods for condition identification; (7) standard and reliable measurement; (8) appropriateness of statistical analysis; and (9) adequacy of response rate. Each item was rated as “yes” (low risk of bias), “no” (high risk of bias), “unclear” (insufficient information), or “not applicable.” Disagreements between reviewers were resolved through discussion with a third reviewer (EK). No studies were excluded based on the risk-of-bias assessment to ensure a comprehensive synthesis of the available evidence, although the quality of evidence was considered during the interpretation of findings. Detailed findings from the risk-of-bias assessment are provided in Table S2, with key methodological limitations synthesized in the Results section.

### Data synthesis and statistical analysis

A meta-analysis of proportions was conducted using the web-based platform MetaAnalysisOnline.com [7]. A random-effects model (DerSimonian–Laird method) was used to pool prevalence estimates, as significant heterogeneity was anticipated due to variations in study populations, microbiological methods, and gestational ages. Pooled prevalence and 95% confidence intervals (CIs) were calculated using the Freeman–Tukey double arcsine transformation to stabilize variances, and estimates were subsequently back-transformed for interpretation. Statistical heterogeneity was assessed using the I^2^ statistic and the Chi-squared test, with I^2^ values of 25%, 50%, and 75% considered indicative of low, moderate, and high heterogeneity, respectively [8]. Publication bias was planned to be assessed visually using funnel plots and tested using Egger's test for asymmetry, provided a sufficient number of studies (typically ≥ 10) were available [20].

To assess the robustness of the pooled prevalence estimates, a leave-one-out sensitivity analysis was performed by iteratively excluding each study and recalculating the summary effect. Given the methodological differences in amniocentesis techniques and microbiological detection methods, sensitivity analysis was planned specifically to identify the source of statistical heterogeneity. Following the main meta-analysis of overall microbial prevalence, we conducted pathogen-specific analyses. For each organism, data were pooled exclusively from studies that reported the presence or absence of that specific pathogen. When prevalence was not explicitly stated, studies were included if their methodology described using detection techniques selective for the organism of interest. To ensure robust and clinically meaningful estimates, microorganisms were selected for pathogen-specific meta-analysis based on predefined criteria: (1) detection in at least two included studies, ensuring consistency across populations; or (2) detection in a single study but with at least three positive samples and documented presence in the amniotic cavity in the literature, ensuring both internal consistency within the study and external validation from prior evidence.

Studies that did not meet the inclusion criteria for the meta-analysis, i.e., case reports or case series with fewer than 10 AFS cases, were nonetheless reviewed to capture the full spectrum of microorganisms potentially associated with AFS.

To assess the impact of amniocentesis technique on microbiological findings, we compared transabdominal and transvaginal samples using a primarily descriptive approach due to the limited number of studies. This included: (1) comparison of the median numbers of microorganisms per positive sample (Mann–Whitney U test); and (2) qualitative assessment of microbial profiles to identify potential vaginal contaminants.

### Certainty assessment

The certainty of evidence for each pooled prevalence estimate was evaluated using the Grading of Recommendations, Assessment, Development and Evaluations (GRADE) framework. Evidence was assessed across five domains: risk of bias (based on JBI checklist ratings), inconsistency (I^2^ statistics and overlap of confidence intervals), indirectness (applicability to the research question), imprecision (width of confidence intervals and sample size), and publication bias (assessed via funnel plot asymmetry when ≥ 10 studies were available). Certainty was categorized as high, moderate, low, or very low according to standard GRADE definitions. Findings are presented in a summary of findings table (Table S3).

## Results

### Study selection and characteristics

**Fig. 1:**
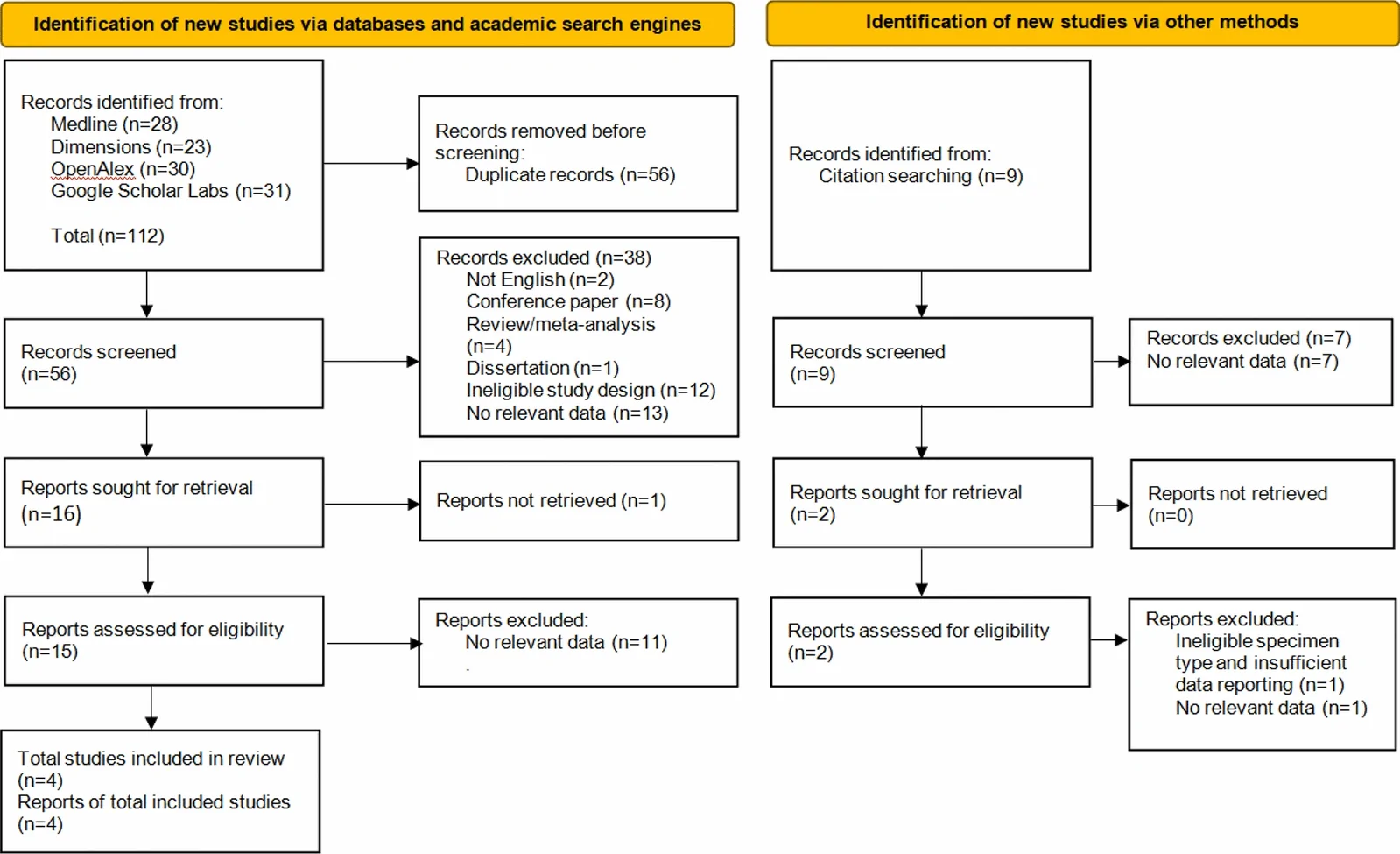
PRISMA flow diagram of study selection

The systematic search identified a total of 112 records from electronic databases and academic search engines. After removing 56 duplicates, 56 records were screened based on titles and abstracts, leading to the retrieval of 15 full-text reports. Eleven studies were subsequently excluded as they did not report the required quantitative data. Searching citation lists identified 9 additional records, but none met the eligibility criteria. Ultimately, four studies comprising 85 subjects were included in the systematic review and meta-analysis [2, 9–11]. The study selection process is detailed in the PRISMA flow diagram (Fig. 1).

The included studies, published between 2005 and 2022, represent diverse clinical populations, including women with preterm labor and intact membranes, asymptomatic high-risk patients with a short cervix, and those with impending delivery. Regarding methodology, three studies utilized transabdominal amniocentesis, while one employed transvaginal sampling. Detection methods primarily relied on standard aerobic/anaerobic and mycoplasma cultures, although one study utilized nested PCR with sequencing confirmation. Detailed characteristics of the included studies are summarized in Table 1. In all included studies, microbiological testing was performed on all women with sonographically detected AFS. No selective testing was applied, minimizing the risk of sampling bias.

**Table 1:** Characteristics of the studies included in meta-analysis

First author Year	Study design	Study population	Specimen collection dates	Gestational age	Type of amniocentesis	Method(s) of detection of microorganisms	No. of positives/total no. (%)	Reference
Espinoza 2005	Retrospective cohort	Women with preterm labor with intact membranes	August 1999–December 2002	Mean ± standard deviation 26 ± 3.8	Transabdominal	Standard aerobic/anaerobic cultures + cultures for genital mycoplasmas	6/18 (33.3%)	[9]
Kusanovic 2007	Retrospective case–control	Asymptomatic high-risk women with short cervix	March 2002–November 2005	Median with interquartile range 21.6 (17.7–23.5)	Transabdominal	Standard aerobic/anaerobic cultures + cultures for genital mycoplasmas	5/23 (21.7%)	[10]
Yoneda 2018	Retrospective cohort	Women with preterm labor with intact membranes	January 2006–November 2015	Median with range 22 (20–29)	Transabdominal	Nested PCR (bacterial 16S rRNA gene, Mycoplasma/ureaplasma-specific, fungal ITS); sequencing confirmation of positive samples	6/19 (31.6%)	[11]
Kusanovic 2022	Cross-sectional	Women with impending delivery (preterm and term)	Not specified	Range 18–40	Transvaginal	Standard aerobic/anaerobic cultures + cultures for genital mycoplasmas	13/25 (52%)	[2]

**Fig. 2:**
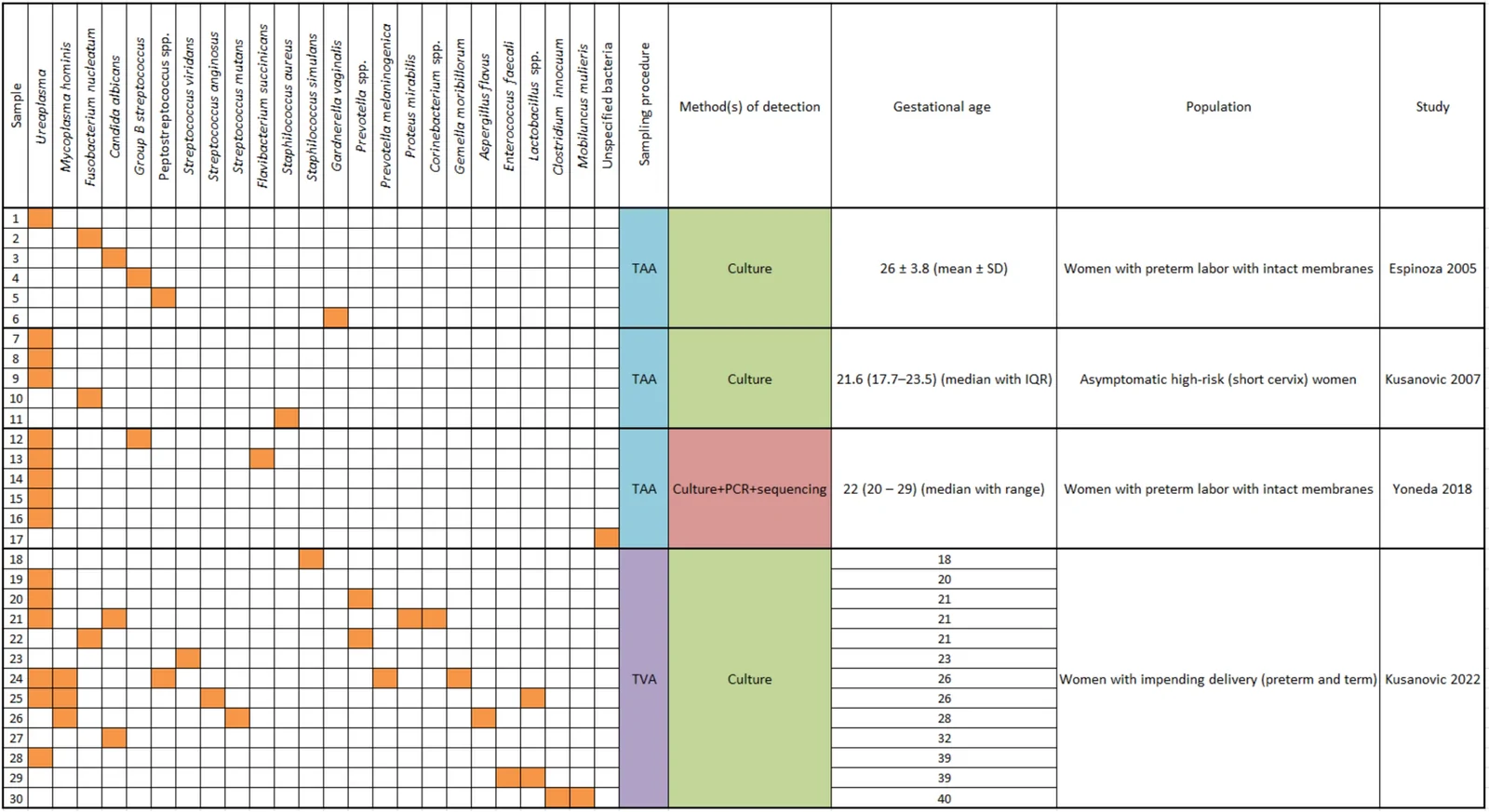
Microbial distribution across positive AFS samples. Each row represents a positive sample and relevant characteristics. Gestational age data that were not reported individually are presented for the amniotic fluid sludge subsets of patients. *TAA* transabdominal amniocentesis, *TVA* transvaginal amniocentesis, *SD* standard deviation, *IQR* interquartile range

To enhance transparency and provide a comprehensive overview of the microbial landscape, a detailed scheme of the microbial distribution across individual positive samples was constructed (Fig. 2). Each row in this figure represents a microbiologically positive sample, aligned with relevant patient and procedural characteristics.

### Risk-of-bias assessment

All included studies provided clear descriptions of their settings and participants and utilized valid, reliable methods for microbial identification. However, overall certainty was affected by small sample sizes and specific methodological variations. In particular, the potential for contamination in one study employing transvaginal sampling was identified as a notable source of bias. Detailed findings from the risk-of-bias assessment are provided in Table S2.

### Pooled overall and pathogen-specific prevalence

**Fig. 3:**
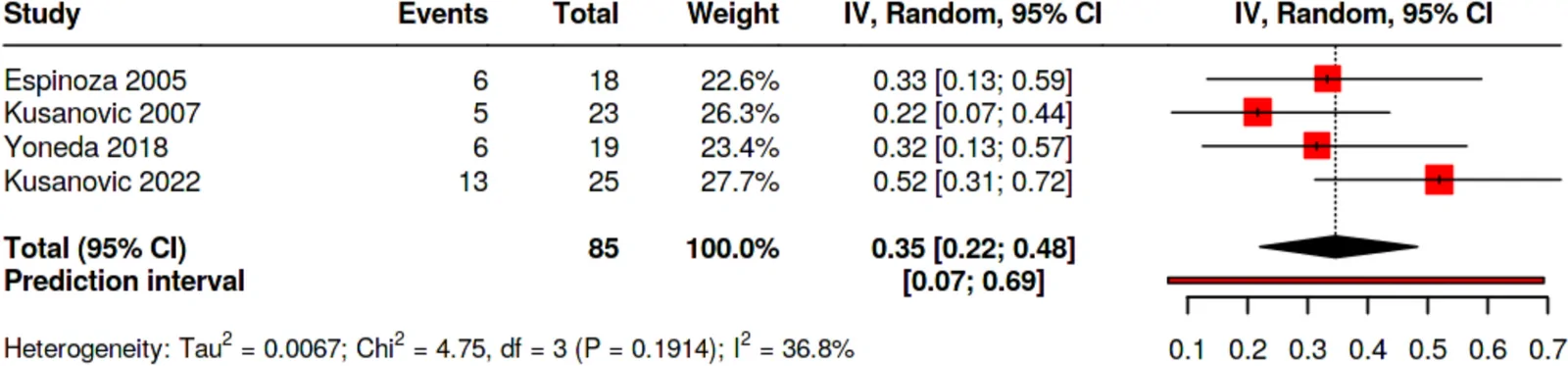
Pooled prevalence of microorganisms (any) in amniotic fluid from women with amniotic fluid sludge

Using a random-effects model with the inverse variance method and Freeman–Tukey double arcsine transformation, the pooled prevalence of any microorganism was 35% (95%CI: 22–48%) (Fig. 3). Pathogen-specific pooled prevalence estimates were calculated for microorganisms meeting predefined criteria. *Ureaplasma* spp. was the most commonly identified microorganism, with a pooled prevalence of 17% (95%CI: 8–27%; Figure S1). *F. nucleatum* followed at 3% (95%CI: 0–9%; Figure S2), and *C. albicans* exhibited a prevalence of 2% (95%CI: 0–8%; Figure S3). Group B Streptococcus (GBS), *Peptostreptococcus* spp., and *M. hominis* each demonstrated a 1% prevalence (95%CIs: 0–6%, 0–6%, and 0–8%, respectively; Figures S4–S6).

Heterogeneity varied across the specific organisms analyzed. Estimates for *F. nucleatum* (I^2^ = 0%, *p* = 0.732), GBS (I^2^ = 0%, *p* = 0.445), and *Peptostreptococcus* spp. (I^2^ = 0%, *p* = 0.568) demonstrated absent heterogeneity. *C. albicans* (I^2^ = 8.5%, *p* = 0.350) and *Ureaplasma* spp. (I^2^ = 20.5%, *p* = 0.287) showed low heterogeneity. Analysis of *M. hominis* revealed moderate heterogeneity (I^2^ = 40.8%; *p* = 0.167), indicating some variability in prevalence estimates—though not statistically significant—and comparable to the overall estimate for any microorganism (I^2^ = 36.8%, *p* = 0.191).

### Microorganisms identified in amniotic fluid sludge: summary of studies not meeting meta-analysis inclusion criteria

Table 2 summarizes case reports, case series, and other non-eligible studies on amniotic fluid sludge that were not included from the meta-analysis, but provide valuable microbiological context. These reports document a diverse array of microorganisms detected in AFS, including *F. nucleatum* [12–14], *C. albicans* [15, 16], *Ureaplasma* spp. [17], and other species [17–19]. Notably, *F. nucleatum* and *C. albicans* were identified across multiple independent reports, corroborating their role as clinically significant pathogens in AFS. Of note, Gauthier et al. [14] detected *F. nucleatum* in all three AFS cases examined. However, this study selectively enrolled women already confirmed to have *F. nucleatum* in amniotic fluid from a larger cohort of preterm labor, and then retrospectively examined oral samples from the women and their partners to trace the bacterial origin. This study provides mechanistic evidence for transmission pathways, but does not reflect prevalence in unselected AFS populations.

**Table 2:** Microorganisms identified in women with amniotic fluid sludge: summary of non-eligible studies

First author Year	Study design	Clinical presentation	Gestational age	Type of amniocentesis	Method(s) of detection of microorganisms	Microorganism(s) detected in amniotic fluid	References
Romero 2007	Case report	Preterm labor, clinical chorioamnionitis	28	Transvaginal	Culture	*Mycoplasma hominis, Streptococcus mutans, Aspergillus flavus*	[18]
Gauthier 2011^a^	Transversal study nested in a cohort (n = 3)	Preterm labor (all 3 with AFS)	18–24	Transabdominal	Culture + Fusobacterium-specific PCR + DNA sequencing	*Fusobacterium nucleatum* in all three women + *Streptococcus viridians* in one women	[14]
Rhalmi 2011	Case report	Cervical insufficiency	23	Transabdominal	Culture	*Fusobacterium nucleatum*	[12]
Himaya 2011	Prospective cohort (microbiological investigation of amniotic fluid in 1 case)	Cervical insufficiency	22	Transabdominal	Culture	*Staphylococcus warneri*	[19]
Paules 2015	Case series (n = 4)	Cervical insufficiency	(1) 21 (2) 22 (3) 24 (4) 23	Transabdominal	Culture	(1) *Fusobacterium nucleatum* (2) Negative (3) Not done (4) *Fusobacterium nucleatum*	[13]
Kusanovic 2018	Case report	Vaginal bleeding and irregular uterine contractility	20	Transabdominal	Culture	*Candida albicans*	[15])
Jung 2020	Case report	Cervical insufficiency	20	Transabdominal	Culture	1st amniocentesis: *Bacteroides ureolyticus, Lachnoanaerobaculum saburreum, Lachnoanaerobaculum umeaense* 2nd amniocentesis: *Streptococcus mitis, Eikenella corrodens, Ureaplasma urealyticum*	[17]
Best 2022	Case report	Cervical insufficiency	22	Transabdominal	Culture	*Candida albicans*	[16]

^a^The study by Gauthier et al. [14] selectively enrolled women already known to have *F. nucleatum* in amniotic fluid from a larger preterm labor cohort, and then retrospectively examined oral samples from the women and their partners to investigate the bacterial origin. The 100% detection rate reflects this selection for the outcome of interest and provides mechanistic evidence for oral-hematogenous or oral-genital transmission, but does not represent prevalence in unselected AFS populations

### Impact of amniocentesis technique on microbial diversity

The impact of amniocentesis technique on microbial diversity was evaluated across four studies. Three employed transabdominal sampling in non-laboring women with preterm labor or cervical insufficiency, while one used transvaginal sampling in women with impending preterm or term delivery. Visual inspection of the microbial distribution (Fig. 2) revealed marked differences between techniques. Transabdominal samples typically contained one, or occasionally two, microorganisms per positive sample. In contrast, transvaginal samples exhibited substantially greater microbial diversity, with multiple organisms frequently detected. Quantitatively, the number of microorganisms per positive sample was significantly higher in the transvaginal group (median = 2, range: 1–5) compared to the transabdominal group (median = 1, range: 1–2; Mann–Whitney U = 49.0, *p* = 0.005).

This difference in diversity was further reflected in the types of organisms identified. Microorganisms typical of normal vaginal microbiota, including *Lactobacillus* species, were detected exclusively in transvaginally collected samples, suggesting possible contamination from the vaginal environment. Notably, *M. hominis* was also detected only in transvaginal samples, which likely contributed to the substantial, though not statistically significant, heterogeneity observed for this organism (I^2^ = 40.8%). The absence of *M. hominis* in transabdominally sampled cohorts suggests that its detection may reflect sampling technique rather than true intra-amniotic prevalence.

The impact of sampling technique extended beyond microbial composition to overall detection rates. The transvaginally sampled study reported a markedly higher prevalence of any microorganism (52%) than the transabdominal studies (21.7–33.3%), which likely accounts for the moderate heterogeneity (I^2^ = 36.8%) in the pooled estimate for overall microbial presence.

**Fig. 4:**
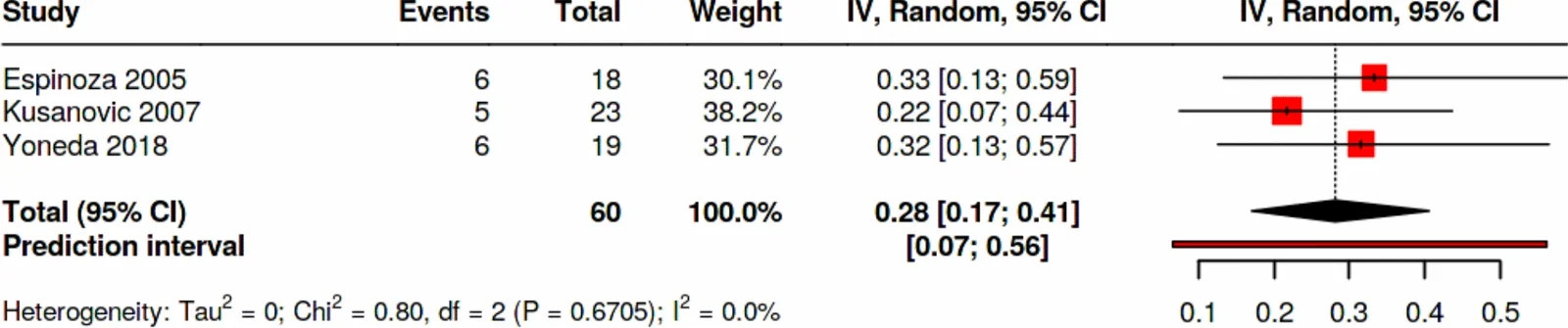
Pooled prevalence of microorganisms (any) in amniotic fluid sludge based on transabdominal sampling only. Exclusion of the transvaginal study resulted in no statistical heterogeneity (I^2^ = 0%), with a pooled prevalence of 28% (95%CI 17–41%)s

To further assess this impact, a leave-one-out sensitivity analysis was performed for the overall microbial prevalence estimate, given its moderate heterogeneity. This analysis confirmed that the pooled prevalence was influenced by the inclusion of the single study utilizing transvaginal amniocentesis [2]. Excluding this study reduced the pooled prevalence from 35% (95%CI: 22–48%) to 28% (95%CI: 17–41%; Fig. 4). Notably, this exclusion also resulted in a complete elimination of statistical heterogeneity, with the I^2^ value dropping from 36.8% to 0.0%. Importantly, despite the shift in the point estimate, the 95% confidence intervals remained largely overlapping, indicating that the presence of microorganisms in approximately one-third of amniotic fluid samples is a relatively stable finding. For pathogen-specific meta-analyses, sensitivity analysis was not warranted due to low or absent statistical heterogeneity and the low number of positive events, with the notable exception of *M. hominis*, which was exclusively detected in the excluded transvaginal cohort.

In summary, this analysis reinforces the critical influence of sampling method on microbiological findings and underscores the need for standardized approaches. Despite these technique-related differences, the low statistical heterogeneity across main pathogen-specific analyses confirms that inclusion of the transvaginal study did not distort pooled prevalence estimates for the primary microorganisms. These findings underscore that while transvaginal sampling may detect additional organisms—likely reflecting contamination—the core prevalence estimates for intra-amniotic fluid remain stable.

### Publication bias

Publication bias was not formally assessed via funnel plot asymmetry or Egger's test, as the meta-analysis included fewer than 10 studies, rendering such methods underpowered to distinguish chance from real asymmetry [20].

### Certainty of evidence

GRADE assessment (Table S3) revealed moderate certainty for the pooled prevalence of any microorganism in transabdominal samples (28%, 95%CI 17–41%), as well as for *Ureaplasma* spp. (17%, 95%CI 8–27%). Certainty was downgraded one level for imprecision due to the small number of participants and wide confidence intervals.

Low certainty was assigned to the overall prevalence estimate including all studies (35%, 95%CI 22–48%) due to serious risk of bias (transvaginal sampling contamination) and imprecision. Estimates for *F. nucleatum* (3%, 95%CI 0–9%), *C. albicans* (2%, 95%CI 0–8%), GBS (1%, 95%CI 0–6%), and *Peptostreptococcus* spp. (1%, 95%CI 0–6%) were also graded as low certainty, primarily due to imprecision from low event rates and wide confidence intervals.

Very low certainty was assigned to *M. hominis* (1%, 95%CI 0–8%), as detection occurred exclusively in the single study using transvaginal sampling, raising serious concerns about contamination bias, compounded by imprecision and moderate inconsistency (I^2^ = 40.8%).

## Discussion

### Principal findings

This meta-analysis provides the first pooled estimates of microbial prevalence in AFS, synthesizing data from 85 subjects across four studies. While the overall microbial prevalence was 35% (95%CI: 22–48%), sensitivity analysis showed that this estimate was influenced by the sampling technique. Excluding the study utilizing transvaginal amniocentesis reduced the prevalence to 28% (95%CI: 17–41%) and resulted in the resolution of statistical heterogeneity, with the I^2^ value dropping from 36.8% to 0.0%. This drop suggests that sampling technique was a source of statistical variance in this dataset. The 28% estimate was consistent across the three transabdominal studies.

*Ureaplasma* spp. emerged as the predominant pathogen, with a pooled prevalence of 17%, accounting for nearly half of all positive cases. The prevalence of other microorganisms was substantially lower: *F. nucleatum* was detected in 3%, *C. albicans* in 2%, and GBS and *Peptostreptococcus* in 1% each. Methodological diversity regarding sampling technique likely contributed not only to the moderate heterogeneity observed for overall microbial presence, but also to that observed for *M. hominis* (I^2^ = 40.8%). Although formal sensitivity analyses were not performed for individual microorganisms due to the low number of events, the fact that *M. hominis* was detected exclusively in the study employing transvaginal amniocentesis suggests that its detection reflects vaginal contamination rather than true intra-amniotic prevalence. These findings underscore the critical importance of utilizing transabdominal amniocentesis for future microbiological investigations of AFS and intra-amniotic infection in general.

Although the included studies enrolled different clinical populations, including preterm labor with intact membranes, asymptomatic short cervix, and impending delivery, the transabdominal cohort showed no statistical heterogeneity with an I^2^ of 0%. This consistency suggests that the microbial profile of AFS is similar across these high-risk groups, supporting the generalizability of our findings.

The included studies evaluated microbiological findings within AFS-positive populations and did not include non-AFS comparator groups. Therefore, our results estimate microbial prevalence among women with AFS and should not be interpreted as quantifying the association between AFS and intra-amniotic infection.

### Key microbial pathogens in AFS

Infection is supposed to play a central role in AFS, which represents a tangible manifestation of the host immune response to microbial invasion. AFS consists primarily of dense inflammatory cell aggregates resulting from intra-amniotic inflammation—a process often, but not always, linked to infection. Our meta-analysis confirms that detectable microorganisms are present in a substantial proportion of AFS cases, with *Ureaplasma* spp. emerging as the predominant pathogen.

*Ureaplasma* spp. are the most frequently identified microorganisms in women with intra-amniotic infection [21]. These bacteria are common commensals of the female genitourinary tract, colonizing 5–20% (non-pregnant women) and 20–90% (pregnant women) [22], but become pathogenic upon ascending into the amniotic cavity [23, 24]. Consistent with the broader literature on intra-amniotic infection [21, 25], *Ureaplasma* spp. were the most frequently identified microorganisms in our analysis, detected in 17% of samples and accounting for nearly half of all positive cases.

Several factors likely explain the predominance of *Ureaplasma* spp. in amniotic fluid sludge. First, it is a very common colonizer of the female genital tract. The high baseline prevalence means that it is the most likely organism to be in a position to ascend into the uterus. *Ureaplasma*, residing in the cervicovaginal area, is perfectly positioned for this route. It can cross intact membranes, though the risk is higher with membrane rupture [23, 25].

Furthermore, *Ureaplasma* spp. possesses unique biological properties that favor survival in the amniotic cavity. Fetal urine, a major component of amniotic fluid in the second and third trimesters, contains urea—a substrate hydrolyzed by the potent urease enzyme of *Ureaplasma* spp. into ammonia and carbon dioxide, providing the bacterium with a readily available energy source [25]. Beyond metabolic adaptation, *Ureaplasma* spp. can evade host immunity while simultaneously triggering a potent inflammatory cascade. Its membrane components are recognized by amniotic macrophages, inducing massive release of pro-inflammatory cytokines (IL-1β, IL-6, TNF-α). This "cytokine storm" recruits neutrophils into the amniotic cavity, where they aggregate with bacteria and cellular debris to form the visible sludge [25].

Biofilm formation seems an attribute of microorganisms involved in AFS. With the use of scanning electron microscopy and fluorescence in situ hybridization, bacteria and extracellular matrix were identified in AFS, confirming it as a microbial biofilm in intra-amniotic infection [4]. Biofilm structure makes the infection much more resilient and difficult to eradicate, contributing to the chronic, smoldering inflammation seen in sludge [26]. Evidence suggests that *Ureaplasma* spp. can form biofilms, although studies evaluating biofilm forming capacities of *Ureaplasma* spp. are scarce [27–29].

*F. nucleatum* emerged as the second most common microorganism detected in amniotic fluid sludge, though its prevalence (3%) was substantially lower than that of *Ureaplasma* spp. (17%). This opportunistic oral anaerobe is associated with periodontal and endodontic diseases and is increasingly recognized as a pathogen in diverse systemic conditions, including cardiovascular disease, inflammatory bowel disease, various cancers (oral, gastrointestinal, genitourinary), rheumatoid arthritis, Alzheimer’s disease, and Lemierre’s syndrome [30]. Furthermore, there is a substantial body of evidence indicating involvement of *F. nucleatum* in adverse pregnancy outcomes, such as chorioamnionitis, PTB, stillbirth, neonatal sepsis, and preeclampsia [31]. Unlike typical genital pathogens, *F. nucleatum* reaches the uterus primarily via hematogenous transmission from the maternal oral cavity, as strains isolated from the placenta most often match those from the mother’s mouth rather than the genital tract [14]. Pregnancy-associated gingivitis facilitates this translocation [32].

*F. nucleatum* possesses several virulence factors that promote colonization, dissemination, and host immune activation. The bacterium is highly adhesive and invasive, capable of coaggregation with other bacterial species and invasion of host cells. The key virulence factor, FadA adhesin, binds to cadherins (VE-cadherin on endothelium, E-cadherin on epithelium), disrupting cell junctions and increasing tissue permeability—facilitating both its own systemic spread and that of other bacteria [30]. Once in placental tissue, *F. nucleatum* triggers a pro-inflammatory response via the TLR4 pathway, contributing to fetal loss and other complications [31].

Within the oral cavity, *F. nucleatum* is a key constituent of subgingival dental plaque biofilms, acting as a critical "bridging" organism between early and late bacterial colonizers [33]. While dental plaque biofilms maintain a balanced composition in health, disease shifts the equilibrium toward tissue destruction, with *F. nucleatum* proliferating in this pathogenic environment. The organism has five known subspecies, four of which are capable of biofilm formation [33].

The pathogenic role of *F. nucleatum* in systemic diseases is often underestimated due to difficulties in culturing this fastidious obligate anaerobe, which requires highly specific growth conditions [34]. Consequently, it frequently underlies "culture-negative" infections. Its prevalence in various systemic conditions has largely been elucidated through culture-independent methods, including 16S rRNA gene sequencing and nucleic acid amplification techniques.

Of note, the case series by Paules et al. [13] —although excluded from our meta-analysis due to its small sample size and case report design—offers important supportive evidence for the role of *F. nucleatum* in AFS. It should be mentioned that the high detection rate reported by Gauthier et al. [14]—where *F. nucleatum* was identified in all three AFS cases—must be interpreted in light of their study design. The authors selectively enrolled women already known to have intra-amniotic *F. nucleatum* from a preterm labor cohort, and then traced the origin to oral samples from the women or their partners. This approach provides compelling mechanistic support for oral-hematogenous or oral-genital transmission pathways but does not reflect the prevalence of *F. nucleatum* in unselected AFS populations, which our meta-analysis estimates at 3%.

*C. albicans* is a direct causal agent in amniotic fluid sludge formation, initiating intra-amniotic infection and inflammation via an ascending route, which is supported by direct visualization of fungal hyphae within sludge samples, in vitro evidence of *C. albicans* penetrating intact fetal membranes, and animal models demonstrating severe intra-amniotic inflammation following *C. albicans* exposure [15, 16, 35–37]. Risk factors facilitating *Candida* ascension include cervical cerclage and intrauterine device conception [15]. Fungal invasion triggers a robust maternal and fetal inflammatory response, characterized by pro-inflammatory cytokine release (IL-6, IL-8, TNF-α) and neutrophil recruitment into the amniotic cavity [37].

*C. albicans* is notorious for forming biofilms, which pose a major clinical challenge due to their inherent resistance to antifungal therapy, structural complexity, and role in device-related infections [38]. The visible sludge represents an accumulation of inflammatory material comprising *C. albicans*, neutrophils, pus, cellular debris, and fungal biofilm matrix. Microscopic analysis confirms fungal hyphae and elevated white blood cell counts within the sludge, substantiating *C. albicans* as the causative pathogen [15]. AFS associated with *C. albicans* is thus conceptualized as a microbial biofilm, where the fungus adheres to surfaces and proliferates, forming a structured community of yeast cells and hyphae.

The presence of *C. albicans*-associated AFS signifies microbial invasion of the amniotic cavity and correlates with severe complications, including acute histological chorioamnionitis [35]. This condition is strongly linked to spontaneous preterm labor, preterm delivery, and elevated rates of neonatal morbidity and mortality. Importantly, transabdominal collection and analysis of AFS permits specific diagnosis of C. albicans infection, enabling targeted antifungal treatment and potentially improving pregnancy outcomes [15, 16].

The pooled prevalence of GBS and *Peptostreptococcus* spp. was 1% each. These are well-established pathogens in intra-amniotic infection; however, while GBS is a leading cause of intra-amniotic infection and associated pregnancy and neonatal complications [39], *Peptostreptococcus* is part of the broader microbial landscape in infectious morbidity [40]. Whereas these bacteria are important causes of intra-amniotic infection, their involvement in AFS formation is poorly documented.

Notably, *M. hominis*, which is often mentioned among the most common bacteria in AFS, was detected exclusively in samples obtained transvaginally in laboring women. This finding strongly suggests that *M. hominis* detection in AFS may reflect contamination from the vaginal microbiota during transvaginal sampling rather than intra-amniotic colonization. These observations underscore the critical importance of standardizing sampling methods in future research to reliably distinguish true intra-amniotic infection from contamination.

Contrary to theoretical concerns about PCR overestimation, our data showed no systematic difference between PCR and culture prevalence estimates. The PCR-based study yielded results nearly identical to culture-based studies using transabdominal sampling. The highest prevalence was observed in a culture-based study with transvaginal sampling. In this dataset, sampling technique appeared to be a more important source of heterogeneity than diagnostic modality. Although PCR and culture are not biologically equivalent, our transabdominal cohort showed remarkably consistent prevalence estimates regardless of detection method. However, only one PCR-based study met the inclusion criteria, precluding a formal subgroup analysis stratified by diagnostic modality. Future studies using both culture and PCR within the same cohort are needed to directly compare these modalities in AFS.

### "Sterile" intra-amniotic inflammation

While infection is considered a primary driver of AFS, a substantial subset of AFS cases (approximately two-thirds to three-quarters of cases) tested negative for microorganisms using standard culture and even molecular methods. Potential triggers in cases of "sterile" intra-amniotic inflammation (intra-amniotic inflammation without detectable microbes) may include failure of microbiological diagnosis, viral infections, and DAMPs released from cellular stress or senescence.

Diagnostic failure may occur under several circumstances. Fastidious organisms—including key AFS-associated microorganisms such as *Ureaplasma* spp., *F. nucleatum*, and *C. albicans*—require specialized culture media and/or prolonged incubation for detection. Additionally, a low microbial burden may fall below the threshold of standard diagnostic assays. Furthermore, when molecular techniques are employed, the chosen method must provide appropriate coverage of potential pathogens; for example, broad-range bacterial PCR targeting the 16S rRNA gene will not detect fungi unless fungal-specific assays are also performed.

Beyond diagnostic limitations, viruses represent another potential trigger of "sterile" intra-amniotic inflammation. Although far less common than ascending bacterial infection, viruses can reach the maternal–fetal interface hematogenously and cause chorioamnionitis. A recent review by Cruz-Holguín et al. [41] introduced the concept of viral "collateral damage" to the placenta, whereby adverse outcomes such as miscarriage and PTB can occur even without vertical transmission. This effect is time-dependent: early infection disrupts implantation, while later infection triggers a pro-inflammatory cytokine storm and placental immune dysregulation, leading to specific histopathological findings including fibrin deposits, calcification, and hemorrhage [41]. Several specific viruses have been implicated in these placental pathologies. Adenovirus DNA was detected significantly more often in preterm placentas (40.8%) than term placentas (20.5%) and was strongly associated with histological chorioamnionitis, particularly before 33 weeks and following seasonal respiratory patterns [42]. Cytomegalovirus, a well-established congenital pathogen, can invade the maternal–fetal interface and induce inflammatory placental damage contributing to adverse outcomes [43]. Other viruses, including human herpesvirus 6, parvovirus B19, and Epstein–Barr virus, have been isolated from amniotic fluid, suggesting potential involvement in intra-amniotic inflammation [44]. However, evidence supporting these as primary causes of chorioamnionitis remains extremely limited.

Sterile inflammation may also be triggered by endogenous "danger signals" rather than infection, playing a crucial role in both normal reproduction and pregnancy complications. Key initiators are DAMPs (or alarmins), such as HMGB1, cell-free fetal DNA, uric acid, and IL-1α, released during cellular stress or death. These molecules activate pattern recognition receptors like Toll-like receptors abundantly expressed in gestational tissues, triggering a pro-inflammatory cytokine response that links placental dysfunction to major complications, including preterm labor, preeclampsia, and fetal growth restriction [45].

Accumulating evidence indicates that AFS can arise from sterile inflammation rather than infection alone. Studies have documented AFS in patients with negative cultures and even highly sensitive molecular methods, yet with elevated intra-amniotic inflammatory markers [11]. In such cases, the immune system recognizes endogenous threats, potentially arising from placental dysfunction or maternal–fetal rejection, triggering inflammation and sludge formation in the absence of identifiable bacteria or fungi [11]. This distinction has important clinical implications, as sterile inflammation may require different management than infection-driven processes, such as immunomodulatory rather than antimicrobial therapy.

### Strengths and limitations

This meta-analysis has several key strengths. To our knowledge, it represents the first systematic synthesis of microbial prevalence in AFS. A major strength is the sensitivity analysis, which identified transvaginal sampling as a source of statistical heterogeneity in this dataset, providing a prevalence estimate of 28% for transabdominal cohorts. Furthermore, the transparent presentation of individual sample-level data (Fig. 2) enhances interpretability, allowing for a clear distinction between intra-amniotic infection and probable vaginal contamination (e.g., *Lactobacillus*). An additional strength of this study is the comprehensive review of non-eligible records, which served as an external validation of our meta-analysis. The high degree of concordance between our pooled estimates and the microbial patterns observed in independent case reports suggests that our findings capture the main microbial patterns reported in the literature on AFS.

Several limitations merit acknowledgment. First, the small number of studies (n = 4) and subjects (*n* = 85) limited estimate precision and precluded formal publication bias assessment. The certainty of evidence, as assessed by GRADE, ranged from moderate to very low across outcomes. While the consistent findings for *Ureaplasma* spp. and for transabdominal samples provide moderate certainty, the low to very low certainty for other estimates underscores the need for larger, methodologically rigorous studies to confirm these preliminary findings. However, the complete elimination of heterogeneity in sensitivity analysis demonstrates the stability of our findings. Second, variability in microbiological methods (culture vs. PCR) across studies may have influenced detection rates.

Second, microbiological methods varied across studies (culture vs. PCR). However, in our dataset, this variability did not translate into statistical heterogeneity: after excluding the transvaginal study, the three transabdominal studies (two culture, one PCR) showed no heterogeneity (I^2^ = 0%), and the PCR-based study yielded prevalence estimates similar to the culture-based studies. Nevertheless, only one PCR-based study met the inclusion criteria, which limits our ability to formally assess whether diagnostic modality influences pooled estimates.

Third, two included studies (Espinoza 2005 and Kusanovic 2007) originated from the same institution with partially overlapping study periods in 2002. Although the clinical populations were distinct, with preterm labor and intact membranes versus asymptomatic short cervix, we cannot completely exclude the possibility of some patient overlap. If overlap occurred, our pooled estimates might slightly overstate precision. However, the consistency of findings across all transabdominal studies, with an I^2^ of 0%, suggests that any potential overlap did not materially affect the conclusions.

Fourth, inclusion of only one study using transvaginal sampling limits generalizability, though its statistically significant impact on microbial diversity was clear. Fifth, the absence of individual participant data precluded adjustment for confounders such as cervical length or antibiotic exposure.

Sixth, without access to subscription databases (Embase, Scopus, Web of Science), our search was limited to free resources (MEDLINE, Dimensions, OpenAlex). While we supplemented with reference screening and AI-assisted searching, studies indexed exclusively in subscription databases may have been missed. However, given our highly specific research question and that all included studies were identified through free databases, the risk of missing pivotal studies is low.

These limitations underscore the need for larger, methodologically standardized prospective studies to validate our findings.

Finally, the review was not prospectively registered in PROSPERO. This limitation stems from the exploratory feasibility assessment we conducted prior to the full review, as it was initially uncertain whether sufficient data existed for a meaningful meta-analysis. Only after confirming that pooled estimates could be generated did we proceed with a full PRISMA-compliant review using a pre-specified internal protocol. While prospective registration is ideal, this sequential approach—feasibility assessment followed by rigorous synthesis—was appropriate given the exploratory nature of this question and the limited evidence base. Nevertheless, the lack of PROSPERO registration is acknowledged as a limitation.

## Conclusion

Approximately one-third of amniotic fluid sludge (AFS) cases are associated with detectable microorganisms: 35% overall and 28% in cohorts sampled by transabdominal amniocentesis. *Ureaplasma* spp. emerged as the predominant pathogen, accounting for nearly half of all positive detections. In this dataset, excluding the transvaginal study eliminated statistical heterogeneity (I^2^ decreased from 36.8% to 0.0%), suggesting that sampling technique may be a source of variability and that transvaginal collection may introduce contamination. However, this finding is based on a single study and requires confirmation. Future microbiological studies of AFS should preferentially use transabdominal amniocentesis with rigorous contamination controls and standardized, sensitive microbiological methods.

Although infection remains an important driver of AFS, the majority of cases are microbiologically “sterile,” highlighting the likely role of intra-amniotic inflammation triggered by endogenous danger signals, viruses, or other non-bacterial agents. Larger, methodologically standardized prospective cohorts are needed to clarify the prognostic significance of specific pathogens and to inform targeted interventions aimed at improving pregnancy outcomes in women with AFS.

## Supplementary Information

Below is the link to the electronic supplementary material.Supplementary file1 (DOCX 569 KB)

## Data Availability

All individual-level data are presented in Fig. 2 of the manuscript.
