# Supplementary material for: The microbiology of amniotic fluid sludge: a systematic review and meta-analysis
Source: Arch Gynecol Obstet. 2026 Jun 19;313(1):260. doi: 10.1007/s00404-026-08492-2 (PMC13526716; doi:10.1007/s00404-026-08492-2)
Supplement: Supplementary file 1 [file 404_2026_8492_MOESM1_ESM.docx]

**Supplementary material**

**The microbiology of amniotic fluid sludge: A systematic review and meta-analysis**

Elena Shipitsyna^1^, Olga Pachulia^2^, Vladislava Khalenko^2^, Ekaterina Kopteeva^2^, Anastasiya Sazonova^2^, Natalya Zhestkova^2^, Olesya Bespalova^2^

^1^Department of Medical Microbiology, D.O. Ott Research Institute of Obstetrics, Gynecology and Reproductive Medicine, St. Petersburg, Russia

^2^Department of Obstetrics and Perinatology, D.O. Ott Research Institute of Obstetrics, Gynecology and Reproductive Medicine, St. Petersburg, Russia

**Supplementary Table S1. The completed PRISMA checklist**

| Section and topic | Item | Checklist item | Location in manuscript |
| --- | --- | --- | --- |
| **TITLE** |  |  |  |
| Title | 1 | Identify the report as a systematic review | Title: "The microbiology of amniotic fluid sludge: A systematic review and meta-analysis" |
| **ABSTRACT** |  |  |  |
| Abstract | 2 | See PRISMA 2020 for Abstracts checklist | Abstract (pages 1-2) |
| **INTRODUCTION** |  |  |  |
| Rationale | 3 | Describe the rationale for the review in the context of existing knowledge | Introduction, paragraphs 2-4 |
| Objectives | 4 | Provide an explicit statement of the objective(s) or question(s) the review addresses | Introduction, final paragraph |
| **METHODS** |  |  |  |
| Eligibility criteria | 5 | Specify the inclusion and exclusion criteria for the review | Methods / Eligibility criteria |
| Information sources | 6 | Specify all databases, registers, websites, organisations, reference lists, and date last searched | Methods / Study design and search strategy (MEDLINE, Dimensions, OpenAlex, Google Scholar Labs; searched to February 11, 2026) |
| Search strategy | 7 | Present the full search strategies for all databases, registers and websites | Methods / Study design and search strategy (search strings provided) |
| Selection process | 8 | Specify the methods used to decide whether a study met the inclusion criteria | Methods / Study selection and data extraction (two independent reviewers, third for consensus) |
| Data collection process | 9 | Specify the methods used to collect data from reports | Methods / Study selection and data extraction (standardized pre-piloted form) |
| Data items | 10a | List and define all outcomes for which data were sought | Methods / Data synthesis and statistical analysis (prevalence of any microorganism, pathogen-specific prevalence) |
|  | 10b | List and define all other variables for which data were sought | Methods / Study selection and data extraction (study characteristics, microbiological methods, gestational age, type of amniocentesis) |
| Study risk of bias assessment | 11 | Specify the methods used to assess risk of bias in the included studies | Methods / Risk of bias assessment (JBI critical appraisal checklist for prevalence studies) |
| Effect measures | 12 | Specify the effect measure(s) used in the synthesis | Methods / Data synthesis and statistical analysis (pooled prevalence with 95% CI) |
| Synthesis methods | 13a | Describe the processes used to decide which studies were eligible for each synthesis | Methods / Eligibility criteria; Methods / Data synthesis and statistical analysis (predefined criteria for pathogen-specific meta-analysis; sensitivity analysis for transabdominal cohort) |
|  | 13b | Describe any methods required to prepare the data for presentation or synthesis | Methods / Data synthesis and statistical analysis (Freeman-Tukey double arcsine transformation) |
|  | 13c | Describe any methods used to tabulate or visually display results | Methods / Study selection and data extraction (Figure 2, individual participant-level data matrix) |
|  | 13d | Describe the methods used to synthesize results | Methods / Data synthesis and statistical analysis (random-effects model, DerSimonian-Laird method) |
|  | 13e | Describe any methods used to explore possible causes of heterogeneity | Methods / Data synthesis and statistical analysis (leave-one-out sensitivity analysis; comparison of sampling techniques using Mann-Whitney U test) |
|  | 13f | Describe any sensitivity analyses conducted | Methods / Data synthesis and statistical analysis (leave-one-out sensitivity analysis); Results / Impact of amniocentesis technique on microbial diversity (sensitivity analysis not warranted for pathogen-specific estimates due to low heterogeneity) |
| Reporting bias assessment | 14 | Describe any methods used to assess risk of bias due to missing results in a synthesis | Methods / Data synthesis and statistical analysis (publication bias planned but not performed due to <10 studies); Results / Publication bias |
| Certainty assessment | 15 | Describe any methods used to assess certainty (or confidence) in the body of evidence | Methods / Certainty assessment (GRADE framework) |
| **RESULTS** |  |  |  |
| Study selection | 16a | Describe the results of the search and selection process | Results / Study selection and characteristics (PRISMA flow diagram, Figure 1) |
|  | 16b | Cite studies that might appear to meet the inclusion criteria, but were excluded, and explain why | Results / Study selection and characteristics; Results / Microorganisms identified in AFS: summary of studies not meeting meta-analysis inclusion criteria (Table 2) |
| Study characteristics | 17 | Cite each included study and present its characteristics | Results / Study selection and characteristics (Table 1, Risk of Bias Assessment, Table S2) |
| Risk of bias in studies | 18 | Present assessments of risk of bias for each included study | Results / Risk of Bias Assessment (Table S2) |
| Results of individual studies | 19 | For all outcomes, present for each study summary statistics and effect estimates | Results / Study selection and characteristics (Figure 2, individual sample-level data); Results / Pooled overall and pathogen-specific prevalence (Figures 3, S1-S6) |
| Results of syntheses | 20a | For each synthesis, summarize the characteristics and risk of bias among contributing studies | Results / Study selection and characteristics (Table 1, Risk of Bias Assessment, Table S2) |
|  | 20b | Present results of all statistical syntheses conducted | Results / Pooled overall and pathogen-specific prevalence (pooled prevalence: 35% overall; 17% Ureaplasma; 3% F. nucleatum; 2% C. albicans; 1% GBS, Peptostreptococcus, M. hominis) |
|  | 20c | Present results of any investigations of possible causes of heterogeneity | Results / Impact of amniocentesis technique on microbial diversity (sensitivity analysis excluding transvaginal study: I² 36.8% → 0%, Figure 4; M. hominis detected exclusively in transvaginal study, explaining I² = 40.8%) |
|  | 20d | Present results of all sensitivity analyses conducted | Results / Impact of amniocentesis technique on microbial diversity (leave-one-out sensitivity analysis, Figure 4) |
| Reporting biases | 21 | Present assessments of risk of bias due to missing results | Results / Publication bias (not assessed due to <10 studies); Discussion / Strengths and limitations |
| Certainty of evidence | 22 | Present assessments of certainty (or confidence) in the body of evidence | Results / Certainty of evidence (Table S3) |
| **DISCUSSION** |  |  |  |
| Discussion | 23a | Provide a general interpretation of the results in the context of other evidence | Discussion / Principal findings; Discussion / Key microbial pathogens in AFS |
|  | 23b | Discuss any limitations of the evidence included in the review | Discussion / Strengths and limitations |
|  | 23c | Discuss any limitations of the review processes | Discussion / Strengths and limitations |
|  | 23d | Discuss implications of the results for practice, policy, and future research | Conclusion |
| **OTHER INFORMATION** |  |  |  |
| Registration and protocol | 24a | Provide registration information for the review | Not registered (acknowledged in Methods / Study design and search strategy; Discussion / Strengths and limitations) |
|  | 24b | Indicate where the review protocol can be accessed | Not applicable |
|  | 24c | Describe and explain any amendments to information provided at registration | No registration, therefore no amendments to report |
| Support | 25 | Describe sources of financial or non-financial support for the review | Declarations / Funding |
| Competing interests | 26 | Declare any competing interests of review authors | Declarations / Conflict of interest |
| Availability of data and materials | 27 | Report which of the following are publicly available: data, code, other materials | Declarations / Data availability (all individual-level data presented in Figure 2) |

**Supplementary Table S2**

**JBI Critical Appraisal Results: Microbiology of Amniotic Fluid Sludge**

| Criteria | Espinoza 2005 | Kusanovic 2007 | Yoneda 2018 | Kusanovic 2022 |
| --- | --- | --- | --- | --- |
| 1. Was the sample frame appropriate to address the target population? | Yes | Yes | Yes | Yes |
| 2. Were study participants sampled in an appropriate way? | Yes | Yes | Yes | Yes |
| 3. Was the sample size adequate? | No | No | No | No |
| 4. Were the study subjects and the setting described in detail? | Yes | Yes | Yes | Yes |
| 5. Was the data analysis conducted with sufficient coverage of the identified sample? | Yes | Yes | Yes | Yes |
| 6. Was the condition (microorganisms in AF) measured in a valid and reliable way? | Yes | Yes | Yes | Yes |
| 7. Was the condition (AF sludge) measured in a standard, reliable way? | Yes | Yes | Yes | Yes |
| 8. Was there appropriate statistical analysis? | Yes | Yes | Yes | Yes |
| 9. Was the response rate adequate, and if not, was it managed appropriately? | N/A | N/A | N/A | Yes |
| Overall Appraisal | Include | Include | Include | Include |

NA, not applicable

**Supplementary Table S3. GRADE assessment of evidence for microbial prevalence in amniotic fluid sludge**

| Outcome | № of participants (studies) | Pooled prevalence (95% CI) | Risk of bias | Inconsistency | Indirectness | Imprecision | Publication bias | Overall certainty |
| --- | --- | --- | --- | --- | --- | --- | --- | --- |
| Any microorganism (all studies) | 85 (4 observational) | 35% (22-48%) | Serious^1^ | Not serious^2^ | Not serious | Serious^3^ | Undetected^4^ | ⨁⨁⊝⊝ **LOW** |
| Any microorganism (transabdominal only) | 60 (3 observational) | 28% (17-41%) | Not serious^5^ | Not serious^6^ | Not serious | Serious^3^ | Undetected^4^ | ⨁⨁⨁⊝ **MODERATE** |
| *Ureaplasma* spp. | 85 (4 observational) | 17% (8-27%) | Not serious^7^ | Not serious^8^ | Not serious | Serious^3^ | Undetected^4^ | ⨁⨁⨁⊝ **MODERATE** |
| *F. nucleatum* | 85 (4 observational) | 3% (0-9%) | Not serious^9^ | Not serious^10^ | Not serious | Very serious^11^ | Undetected^4^ | ⨁⨁⊝⊝ **LOW** |
| *C. albicans* | 85 (4 observational) | 2% (0-8%) | Not serious^9^ | Not serious^12^ | Not serious | Very serious^11^ | Undetected^4^ | ⨁⨁⊝⊝ **LOW** |
| GBS | 85 (4 observational) | 1% (0-6%) | Not serious^9^ | Not serious^13^ | Not serious | Very serious^11^ | Undetected^4^ | ⨁⨁⊝⊝ **LOW** |
| *Peptostreptococcus* spp. | 85 (4 observational) | 1% (0-6%) | Not serious^9^ | Not serious^14^ | Not serious | Very serious^11^ | Undetected^4^ | ⨁⨁⊝⊝ **LOW** |
| *M. hominis* | 85 (4 observational) | 1% (0-8%) | Very serious^15^ | Serious^16^ | Not serious | Very serious^11^ | Undetected^4^ | ⨁⊝⊝⊝ **VERY LOW** |

^1^ Risk of bias - serious: One study (Kusanovic 2022) used transvaginal sampling in laboring women, introducing high risk of contamination bias.

^2^ Inconsistency - not serious: I² = 36.8% (moderate), fully explained by sampling technique in sensitivity analysis.

^3^ Imprecision - serious: Total sample size <100; confidence interval width >20%.

^4^ Publication bias - undetected: Formal assessment not possible due to <10 studies; comprehensive search reduces concern.

^5^ Risk of bias - not serious: All three studies used transabdominal sampling with appropriate methods.

^6^ Inconsistency - not serious: I² = 0.0% after excluding transvaginal study.

^7^ Risk of bias - not serious: Detected consistently across both sampling methods; biological plausibility supports true presence.

^8^ Inconsistency - not serious: I² = 20.5% (low); p=0.287.

^9^ Risk of bias - not serious: Detected only in transabdominal studies; no contamination concern.

^10^ Inconsistency - not serious: I² = 0%; p=0.732.

^11^ Imprecision - very serious: Confidence interval includes zero; very low event rate (<5 cases).

^12^ Inconsistency - not serious: I² = 8.5%; p=0.350.

^13^Inconsistency - not serious: I² = 0%; p=0.445.

^14^ Inconsistency - not serious: I² = 0%; p=0.568.

^15^ Risk of bias - very serious: Detected exclusively in transvaginal study; high likelihood of contamination.

^16^ Inconsistency - serious: I² = 40.8% (moderate), attributable to sampling technique.

Supplementary figure S1. Pooled prevalence of *Ureaplasma* spp. in amniotic fluid from women with amniotic fluid sludge

Supplementary figure S2. Pooled prevalence of *Fusobacterium nucleatum* in amniotic fluid from women with amniotic fluid sludge

Supplementary figure S3. Pooled prevalence of *Candida albicans* in amniotic fluid from women with amniotic fluid sludge

Supplementary figure S4. Pooled prevalence of group B streptococcus in amniotic fluid from women with amniotic fluid sludge

Supplementary figure S5. Pooled prevalence of *Peptostreptococcus* spp. in amniotic fluid from women with amniotic fluid sludge

Supplementary figure S6. Pooled prevalence of *Mycoplasma hominis* in amniotic fluid from women with amniotic fluid sludge
